# Non‐clinical evidence for modulating synaptic CSF biomarkers by E2511, a novel small molecule TrkA biased positive allosteric modulator

**DOI:** 10.1002/alz.095071

**Published:** 2025-01-09

**Authors:** Naoki Hiramatsu, Takeyasu Tomioka, Kazunari Sasaki, Satya Saxena, Kristin R Wildsmith, Pallavi Sachdev, Natasha Penner, Luigi Giorgi

**Affiliations:** ^1^ Eisai Co., Ltd, Tsukuba Japan; ^2^ Eisai Inc., Nutley, NJ USA; ^3^ Eisai Ltd, Hatfield United Kingdom

## Abstract

**Background:**

Cholinergic innervation is particularly vulnerable in many neurodegenerative diseases such as Alzheimer’s diseases. Nerve growth factor (NGF) plays a major role in the maintenance and function of cholinergic neurons, and a decrease in trophic signalling by NGF‐Tropomyosin receptor kinase A (TrkA) contributes to cholinergic and synaptic degeneration. E2511 is a novel small molecule TrkA biased positive allosteric modulator showing an increase in specific trophic signalling via direct binding to TrkA with a potential to recover and reinnervate damaged cholinergic neurons. We have shown that multi‐platform global proteomic approaches identified novel candidate PD biomarkers for E2511 using pre‐ and post‐treatment CSF samples from E2511‐A001‐005 (a multiple‐ascending dose study in healthy volunteers). Modulation of proteins related to cholinergic pathway, neurotrophin/Trk signalling and synaptic pathway was demonstrated for E2511 relative to placebo (CTAD2023, ADPD2024). Here we examined the mechanism of each biomarker changes by E2511 by non‐clinical study.

**Method:**

The effect of E2511 on gene expression of candidate PD biomarkers identified using multi‐platform global proteomic approaches was assessed by qPCR in septum, one of the basal forebrain regions and where cholinergic nerves are localized, of normal SD rats treated with once‐daily oral E2511 for 14 days. The effect of cholinesterase inhibitor (ChEI) on gene expression was also investigated in rats treated with TID oral donepezil for 4 days.

**Result:**

E2511 increased gene expression of several candidate biomarkers related to cholinergic pathway, neurotrophin/Trk signalling and synaptic pathway. In contrast, treatment with donepezil which resulted in an increase in ACh levels failed to elicit such effects on the candidate biomarkers. These results suggest that the increase in gene expression related to cholinergic pathway, neurotrophin/Trk signalling and synaptic pathway by E2511 is via NGF‐TrkA signal activation and E2511 has a mechanism of action distinct from ChEIs.

**Conclusion:**

The mechanism of modulating novel candidate PD biomarkers in human CSF by E2511 was clarified by non‐clinical study using rats. The convergence of clinical global proteomics data and non‐clinical data have identified PD biomarkers for E2511 consistent with the proposed mechanism of action. Results are supportive of continued development of a E2511‐specific biomarker panel and suggestive of pathway activation in healthy volunteers.

